# Improving Food Safety Culture in Nigeria: A Review of Practical Issues

**DOI:** 10.3390/foods10081878

**Published:** 2021-08-13

**Authors:** Helen Onyeaka, Osmond C. Ekwebelem, Ukpai A. Eze, Queeneth I. Onwuka, Job Aleke, Ogueri Nwaiwu, Joy Onyinyechi Chionuma

**Affiliations:** 1 School of Chemical Engineering, University of Birmingham, Edgbaston, Birmingham B15 2TT, UK; 2 Faculty of Biological Sciences, University of Nigeria, Nsukka 410001, Nigeria; osmond.ekwebelem.197684@unn.edu.ng (O.C.E.); alekejob@gmail.com (J.A.); 3 School of Life Sciences, Faculty of Health and Life Sciences, Coventry University, Coventry CV1 5FB, UK; ukpai.eze@gmail.com; 4 Department of Food Science and Technology, Abia State University, Uturu 441101, Nigeria; quencyonwuka@gmail.com; 5 Ingenuity Lab, Jubilee Campus, University of Nottingham, Nottingham NG8 1BB, UK; ogueri.nwaiwu@alpha-altis.co.uk; 6 College of Medicine, Lagos State University, Lagos P.M.B. 0001, Nigeria; o_agbara@yahoo.com

**Keywords:** food safety, foodborne illness, consumer demand, producer behavior, food handling, organizational culture

## Abstract

As a developing nation and the most populous nation in Africa, Nigeria has enormous challenges connected with food safety culture. To produce and provide safe, secure and nutritious food, consumers and food businesses must abide by a set of shared values known as food safety culture. In Nigeria, food safety culture is a complex subject due to Nigeria’s heterogeneous and diverse nature, as demonstrated by its over 250 ethnic groups. As Nigeria becomes more urbanized and incomes continue to fluctuate at robust rates, few Nigerians are conscious of food safety issues. In addition, oversight from government regulators around food safety require improvement. Public engagement in food safety issues has not witnessed a promising trajectory in recent years. In this article, we provide a review of the food safety culture in Nigeria and its role and influence on various cases of food safety issues in Nigeria. Of interest to this paper are studies exploring consumer and food handler perceptions and behavior regarding food safety. In addition, keen attention is devoted to areas that are in need of additional research to help address practical and on-the-ground challenges associated with Nigeria’s food safety practices. This article suggests that improving food safety culture in Nigeria requires both applying the best management and communication approaches in different regions and understanding the local food safety practices.

## 1. Introduction

Food safety is one of the most significant public health issues worldwide, particularly in emerging and developing countries [1]. It has also become one of the most challenging social issues that needs to be addressed in most low- to middle-income countries, including Nigeria. The World Health Organization (WHO) developed five crucial components for achieving safer food. The key features of these are: ensuring cleanliness, separating raw and cooked food, cooking thoroughly, keeping food at safe temperatures, and using safe water and raw materials [2]. Food safety culture describes a collection of learned and shared attitudes, values and beliefs that form the foundation of the hygienic behaviors used within a particular food handling environment [3,4]. Sharman et al. defines food safety culture as a long-term paradigm in a food handling organization deeply rooted in beliefs, behaviors and assumptions which impact the food safety performance within the organization [5]. Recently, food safety culture assessments among consumers and food businesses has gained attention from both scientists and food regulatory agencies [6,7,8,9,10]. Despite this merited attention and the efforts made to date to advance and implement food safety management systems (FSMSs), consumer food poisoning and outbreaks are still reported and remain an important cause of human disease [9,10,11,12]. Hence, the focus has shifted from a formal and technical-oriented food safety management systems approach to a more human approach, as mirrored by the emergence of the concept of food safety culture [13,14,15,16]. 

In Nigeria, over 200,000 people die from foodborne illness annually. The economic burden associated with foodborne illnesses is around US$ 3.6 billion per annum [17]. As in most developing countries, meeting the WHO’s five key requirements for achieving safer food has been a struggle in Nigeria where basic amenities, particularly running water and robust sanitary units, are lacking [18]. These gaps in all aspects of the food chain—from the farm to the table—have amplified food safety issues in Nigeria. By the same token, other issues have contributed to poor food safety practices in Nigeria, including the rising population, the disparity in incomes, the extended food supply chains, the constantly evolving demographics, the dearth of education, the food consumption patterns, little or lack of food safety regulation, and other factors that are endemic in places with low levels of economic development [18,19,20]. Unfortunately, most households with low socioeconomic status mainly eat staples that are produced by the informal sector, exposing them to monotonous diets, which further compound issues around food safety culture. Furthermore, opportunistic and profit-driven behaviors, such as food fraud and adulteration and food information asymmetries, have also constantly fueled the fire of food safety concerns in Nigeria [17,18,21].

In Nigeria, fundamental facilities and adequate enlightenment or sensitization on the importance of food safety culture are gravely lacking in many rural and sub-urban regions [22,23,24,25]. Some of the factors that have been identified as contributing to foodborne outbreaks in Nigeria include: methods of cooking; poor food practices such as inadequate refrigeration, prolonged handling and improper reheating of cooked food; and food contamination by commercial or household food handlers [26,27]. Furthermore, the nature of food safety management in Nigeria is evolving, as the amount of ready-to-eat foods sold in the informal sector as street food have gained significant traction in society [17,18]. Ironically, the economic situation of the country has motivated these changing lifestyles. Interestingly Jespersen et al. [9] proposed that by enumerating food safety maturity via a validated triangulation method, food handling organizations can estimate the proportion of their sales that are wasted through the cost of poor quality items. This approach can have positive impacts in the design of specific interventions, which are needed in order to strengthen food safety management and to control activities in an organization [9]. With a looming global food crisis, the current COVID-19 pandemic (caused by SARS-CoV-2), and the ever-increasing Nigerian population [28,29], the subject of food safety should be a focal point of discourse. In light of this, this paper aims to review food safety in Nigeria and evaluate consumer and producer behavior. The factors affecting basic food safety are also highlighted.

## 2. Perception of Food Safety among Consumers, Food Businesses, and Farmers

Generally, two interesting phenomena associated with consumer’s perceptions of food safety and quality have been observed [30]. Firstly, there is a perception among consumers that ready-made meals (a prepackaged meal requiring very little or no preparation besides heating up) are more dangerous than home-cooked meals, and this potential danger can be worsened when new technologies are used. Secondly, there is a perception that familiar risks are less severe than unfamiliar ones [30]. In Nigeria, remarkably, the second phenomenon takes precedence over the first [30]. In addition, consumers and manufacturers possess striking differences in their perceptions of food safety. Consumers are focused on different components such as packaging, taste, and richness in nutrients as critical components of quality, whereas manufacturers consider product design, performance, and forms to be crucial components of food safety [30,31].

As society continues to evolve rapidly due to social innovations, more and more consumers in Nigeria are spending an increasingly significant portion of their income in ways remarkably different from conventional ways of spending. In Nigeria, a significant proportion of the population—mostly low-income earners—are more interested in money-saving and convenience than in food safety, quality and hygiene [32,33]. The preferred food destination for these people (low-income workers, shoppers, travelers, and school children) are food vendors on the street, as the main objective of these consumers is to quench their hunger with little concern for the safety or nutritional quality of such vended street foods [33,34]. Sadly, unhygienic food handling practices have become common among these informal food-selling vendors, as foods are either cooked, baked, or processed in extremely unsanitary environments. While several consumers and food businesses are keen to improve their food safety practices, the appropriate facilities that would help to promote safe food handling practices have been deficient. For instance, many communities and food businesses lack: adequate means of hygienically washing and drying utensils and equipment; lavatories of appropriate hygienic design; adequate facilities for food storage, ingredients, and non-food chemicals; and adequate drainage and waste disposal systems [35,36,37]. The lack of a continuous power supply further hampers the storage of raw agricultural products and processed food. Additionally, poor maintenance culture is another issue for the few communities with some of these facilitates.

Foods sold through these means are pushed around on food-laden carts, wheel-barrows, or specially-designed bicycles from one location to another in order to serve their customers [38]. Others operate out of small stalls and batches or carry the food around on their heads [17,34]. A study observed that most street food vendors stored their cooking utensils in an open basket, cupboard, or on the floor [34]. Another study that assessed food safety practices in a rural community pointed out that the majority of study groups always washed their hands before cooking, always cleaned their cooking utensils before and after use, always cleaned their cooking environment, and always washed their hands after using the toilet [18]. Although these findings seem positive, they may be subject to bias and there is a likelihood that respondents have given socially desirable responses.

Perceptions of food safety practices and quality among Nigerian farmers have also been studied [39,40]. The emerging picture suggests that food processing is still at the crude stage due to lack of modern processing machinery and food damage due to poor transport infrastructure [39,41]. For example, farmers noted that consumers perceive the famous Abakaliki rice from the South Eastern region as unsafe for consumption, because it contains stones, has low nutrition, and is dirty [39,42,43]. However, in recent times, machines for destoning, cleaning, and polishing such rice have improved consumer perceptions and satisfaction. Similarly, vitamin- and iron-rich fruits such as plantain, mango, guava, and banana are perceived as not being completely safe for consumption because vendors of these products allegedly use harmful chemicals (calcium carbide) to induce to ripen [44].

In south-south Nigeria, both literates and illiterate consumers are concerned about the safety and quality of food due to the presence of impurities like stones, pieces of wood, rats dropping in *garri* (a powdery food made from the tuberous roots of the cassava plant), rice and palm oil [45,46,47,48]. To avoid decay due to inadequate storage facilities, farmers quickly harvest and sell fruits, such as bananas, sugar cane, vegetables, and pineapple [42]. Eventually, such farm produce may be sold cheaply in decaying conditions (as ‘*awarawa*’ in the case of tomatoes), thus increasing the risk of food poisoning and infection due to bacterial and fungal contamination. Other farm produce such as maize, cottonseed, groundnut, cocoa, and plantain are dried and kept in bags and tins to ensure safety and to preserve their quality for sale in the future [39]. However, depending on the storage condition and moisture contents, such farm produce may still be prone to fungal growth, resulting in mycotoxin contamination [49].

## 3. Practical Scenarios of Food Safety Issues in Nigeria

Incidences of food safety issues resulting from poisoning and infection, due to poor food safety practices, unhygienic environments, infectious and toxic agents, are rising in Nigeria. There have been countless cases of food safety issues resulting in deaths in the last few years. Despite this worrying scenario, Nigerians have been largely unaware of this silent killer; however, when it strikes it causes a lot of problems. Interestingly, the rising cases of foodborne illness in Nigeria are not only linked to the misuse and abuse of agrichemicals on agricultural products [17], but are also related to people’s poor food safety practices. In this section, we highlight some of the major food safety issues that have rocked the country in the last decade regarding poor food safety culture in Nigeria.

In April 2021, in Kano (one of the biggest commercial states in Nigeria), ten people died and a further 400 were hospitalized after drinking fruit juice that had expired over a year ago [50]. It was believed that the incident was caused by a chemical called dansami [50]. This scenario highlights the poor culture of checking the “use-by” or “best before” dates of foods and ensuring that any food product beyond its “use-by” date should not be consumed, even if it looks and smells good. In March 2021, 25 people died after eating fried meat served at a bar in Alagbole-Akute, Ogun State [51]. Surprisingly, the deaths were classified as ritual killing and were not considered to be a food poisoning issue. The main cause of the death has not been confirmed to date. Clearly, this highlights the lack of an organized system for monitoring food safety issues in Nigeria. In November 2020, in Joi village of Plateau State, around 20 people were treated for acute gastroenteritis after taking *kunu*, a local drink made from millet. It was found that the grains, which were looted from a COVID-19 palliative storage unit, were bought from a petty trader in the community’s market [52].

From July to August 2017, a diarrheic ailment caused by zoonotic bacteria in meat and water resulted in 62 deaths in Okoloke village, Yagba West in Kogi State, a settlement that is predominantly inhabited by Fulani herdsmen [53]. Notably, this outbreak, which was initially called “a strange disease outbreak” was eventually traced from slaughterhouses to neighborhood food canteens, stalls of itinerant food hawkers, and standard decent eateries and restaurants in the community. Again, this scenario highlights the lack of an organized system for monitoring food safety issues, which explains the inability of authorities to ascertain the true nature of the crisis, and thus, the attribution of the deaths to a “strange disease.” In February 2016, thirteen people died, while ten others were hospitalized for food poisoning caused by *Clostridium perfringes* in Abuja Nigeria’s capital [54]. *Clostridium perfringes* is a bacterium that occurs in the soil and contaminates food and, subsequently, the intestinal tract of human beings [55].

In July 2013, no fewer than 30 guests were hospitalized, and eight persons died after eating a suspected poisoned delicacy *Ugba* (an alkaline fermented African oilbean seed), in a christening ceremony in Owerri Imo State [30]. On 4 June 2012, in Kafur Kastina State, 26 people comprising 20 children and six adults suffered severe gastroenteritis after consuming locally made food (*Tuwo*, a maize meal) prepared with treated guinea corn [56]. Unfortunately, this guinea corn was meant for planting and not for human consumption. Again, in one incident in 2011 in Rivers State, 112 people were reported to have been hospitalized after eating beans preserved with pesticides [57]. Likewise, 120 students of a secondary school in Doma, Gombe State, suffered severe gastroenteritis after eating bean cake contaminated by pesticides.

In 2008, in Bekwara Cross River State, 112 people were hospitalized and two children died due to ingestion of *moi-moi* and beans. The *moi-moi* and beans were said to have contained a large dose of highly toxic pesticides [30,58]. The laboratory report from the National Agency for Food and Drug Administration and Control (NAFDAC) revealed that the beans were contaminated with very high concentrations of organophosphates, carbamates, fenithrothion, and chloropyrifos, all of which are highly toxic pesticides. In a different report, over 120 students of Government Girls Secondary School, Doma, Gombe State were hospitalized after consuming beans [58]. Samples of the meal analyzed by NAFDAC showed that the meal contained very high levels of lindane (an organochlorinated pesticide commonly called Gammallin), which may have been used to preserve the beans from ants and pest attacks.

These high-profile food safety issues indicate that the unhygienic and unsafe handling and treatment of food has seriously impacted public health in Nigeria by causing numerous chronic and non-chronic diseases. Shockingly, the root causes in most of the food safety issues highlighted above were never identified, deeply investigated, and/or communicated to the public in a detailed manner. This highlights the regulatory failures to combat the current food safety problems persisting in Nigeria.

## 4. Attitude towards Food Safety in Nigeria

All the scenarios highlighted above suggest that food safety measures have been intentionally or unintentionally ignored by most food businesses and consumers alike in Nigeria. However, other factors such as regulatory failures, food prices, choice of products, lack of consumer information, and educational and cultural influences may also be responsible for Nigeria’s existing food safety issues. It is common knowledge that regulators focus on big manufacturers, whereas small and artisanal food processors that make the majority of foods consumed by a large number of the population, are ignored. Nigeria is a heterogeneous society with over 250 ethnic groups [59,60]. As such, where people come from plays the biggest role in their food safety practices, because each person has their own food safety practices based on their traditional background. For instance, in Nigeria, popular food businesses—informal food vendors—are mainly owned by and employing people from diverse ethnic backgrounds. Therefore, if conventional food safety culture values are not instilled in the employees, employees from various ethnic and cultural backgrounds would likely perform food safety practices according to their traditions. Without a doubt, this could negatively impact the level of food safety and hygiene.

It is important also to note that some informal food vendors want to improve the food safety culture in their businesses [61,62,63]. However, they have been unable to do so due to financial constraints and/or having hired temporary staff, thereby experiencing challenges in instilling conventional food safety culture among their employees. Various studies have assessed the personal practices and hygiene of food business and the knowledge of food safety cultures among consumers and informal food vendors [18,33,38,64,65,66,67,68]. Interestingly, these studies concluded that further research is still needed in order to quell Nigeria’s poor food safety culture. Some studies have evaluated the knowledge of food safety protocols by food handlers in Southeast Nigeria [38,64]. Unsurprisingly, these studies revealed a lack of knowledge of pathogens and hygienic food safety practices relevant to tropical environments [38,64]. What was somewhat surprising was the lack of knowledge around how allowing a sick person to cook or handle food could expose others to risk and/or result in foodborne illness. Likewise, another study revealed a general lack of knowledge about food safety, contamination, poisoning, control measures, and hygiene practices among formal and informal food vendors in Garki, Abuja [65]. Fortunately, it is feasible and promising to strengthen food safety culture and knowledge and behaviors of food handlers within food handling organizations [69]. There are some strategies that managers, owners, and directors can employ to promote a proactive food safety culture. Some of these strategies include: supplying appropriate infrastructure; creating reliable food safety management systems; and being understanding of employees’ fatigue, job difficulty, and employees’ dissatisfaction within the organization [69]. Interestingly, just as other such as managerial commitments can affect employees’ behavior, intentions, and morale towards food safety practices, these aforementioned factors can also affect food safety and the climate within a food handling organization [69].

These reports have raised questions: could the educational level of food vendors or employees be a factor in the poor food safety culture in Nigeria? Although this question has not been directly answered, a study conducted in eastern Nigeria—one of the regions with the highest literacy rates in Nigeria [70]—reported that foods were unhygienically handled and kept in unsanitary conditions [66]. In this region, it is a common sight to observe factors that contribute to the microbial load of meat, such as constantly handling meat with bare, unwashed hands and the presence of flies. There is also a general lack of understanding among food handlers and consumers around how Nigeria’s hot and humid environment offers bacteria and fungi the ideal conditions for growth [66,67]. Leptospirosis, a bacterial infection of the blood, has been detected as a common disease among abattoir workers in Abuja, Benue, Plateau, and Nassarawa States [53]. In recent years, social media has influenced Nigerian’s attitudes towards food safety. Previously, anonymous posts on social media about food quality have attracted much traffic. Strangely, one of these posts advised consumers against eating or purchasing meat that did not attract flies. As claimed in this post, the lack of flies on meat is an indication that lethal substances were used in the preservation of the meat. The confiscation of a truckload of chickens considered unsafe for consumption by the customs service was cited as veritable proof in this anonymous social media post.

Another study reported the presence of intestinal parasites and bacteria in the stools of food handlers in the Federal Capital Territory [68]. In most urban cities in Nigeria, market gardening allows for the provision of fruits and vegetables all year round. However, these farmers use human and animal feces and wastewater for irrigation in such farms, posing a high risk to the consumers of such fruits and vegetables. In a survey of the different vegetables and fruits harvested in Ebonyi State, South Eastern Nigeria, it was reported that over 40% of the 250 fruits and vegetables were contaminated by pathogenic geohelminths [71], including *Ascaris lumbricoides*, *Strongyloides stercoralis*, *Trichuris trichiura* and *Enterobius vermicularis*. Again, this highlights the need to train farmers and food handlers in food safety practices and to encourage regular check-ups for active illness among staff in informal and formal food businesses.

Developing a food safety culture toolkit will help food-handlers, particularly those with a lower level of education, to improve their poor personal hygiene and help them detect potential sources of infections. Importantly, this toolkit should be simple, not too wordy or repetitive and with an uncomplicated title. The unwillingness of Nigerians to learn lessons from previous food safety issues has raised concerns in recent times. This poor attitude towards food safety culture was also observed in a study conducted in the Are-Ekiti rural community in Ekiti State, southwest Nigeria. This study showed that most households still preferred sun drying for food preservation, despite the high prevalence of Lassa fever in the community [18]. Aside from the unhygienic food preserving methods in this community, the methods of waste disposal and sources of drinking water were identified as potential threats to health and food safety [18]. This situation demonstrates a lack of understanding among locals that refuse dumps serve as a habitat for organisms responsible for typhoid, infantile diarrhea, and cholera in humans [72].

## 5. Food Safety Regulatory and Legislative Framework in Nigeria

The food safety regulatory framework in Nigeria currently functions as a sectoral Food Safety Control System involving multiple agencies. In Nigeria, the onus to implement food safety acts is shared between the three levels of government: namely, federal, state, and local government bodies. The key institutions at the federal level that are responsible for facilitating food safety are the Ministries of Health, Environment, Science and Technology, Agriculture, and Trade and Investment. In conjunction with these ministries are notable agencies under them, such as the National Agency for Food Drug Administration and Control (NAFDAC), Nigerian Institute of Food Science and Technology (NIFST), Nigeria Customs Service, National Biotechnology Development Agency (NABDA), Standards Organization of Nigeria (SON), National Agricultural Seeds Council, Consumer Protection Council, and the National Biosafety Management Agency. NAFDAC, one of the agencies under the Federal Ministry of Health, plays a superior role in regulating and controlling the distribution, sale, and use of food. Equally, the specific role of the Federal Ministry of Environment is intended to ensure that environmental food contaminants, organic pollutants, environmental pollution and waste disposal are adequately controlled. The Federal Ministry of Trade and Investment takes the primary role of maintaining international trade in safe food and promoting the World Trade Organization’s (WTO) sanitary and phytosanitary guidelines in Nigeria.

At the state level, there are state ministries of health and agriculture. Additionally, at the local government level, the Local Government Departments of Health and Agriculture are responsible for enforcing food safety acts and regulating the informal food vendors (street food sellers), catering organizations, local abattoirs, and markets [73]. The food safety challenges in Nigeria have led to the enacting of various legislative acts such as the NAFDAC Act 57 [73]. Generally, the NAFDAC Act 57 has become the foremost Act for regulating food safety in Nigeria. In this Act, NAFDAC provides guidelines for monitoring food irradiation, additives, marketing of food products for infant and young children, fortification regulations, cocoa products regulations, milk and dairy products regulations, pre-packaged food (labelling) regulations, and pesticide registration regulations. Unfortunately, under the NAFDAC Act no provisions or regulations were provided around traceability requirements for food handlers and food business operators in Nigeria [74]. This highlights the need for the amendment of the Act in order to ensure that food business owners and handlers can identify and trace people that they have supplied or sold food to. Although traceability does not necessarily make food safer, it can serve as a risk control tool that can be used in mitigating food safety problems in Nigeria.

In relation to cleaning abattoirs and the environment, most Nigerian states have state sanitation laws, such as laws for the State Waste Management, State Rural Water Supply and Sanitation Agency, State Environmental Sanitation Authority, State Rural Water and Sanitation Agency Laws, State Environmental Protection, State Environmental Pollution Control, and Environmental and Waste Management. While these pieces of legislation are related to food safety, food safety regulation is still not adequate. This realization motivated the development of the National Policy on Food Hygiene and Safety (NPFS) in 2000, as well as the incorporation of the National Policy of Food Hygiene and Safety into the National Health Policy in 2010 [75]. The primary objective of this policy was to achieve and maintain a high level of food hygiene and safety practices among consumers, food handlers, and food businesses in Nigeria, as a sustainable means of promoting health, controlling foodborne diseases and minimizing the risk of diseases due to poor food safety practice [75]. Equally, the NPFS seeks to coordinate all existing laws for the regulation of food safety practices in Nigeria and revamp existing food control systems at the different levels of government [76]. By implementing the National Policy on Food Safety (NPFS), Nigeria has successfully developed its food safety policy. However, this policy is still at the implementation stage. As a result, there are to date no reports evaluating its effectiveness and success.

## 6. Limitations in Promoting Food Safety Culture in Nigeria

Issues around food preparation, safety, sale, and the consumption of street foods in inappropriate places are on the rise in Nigeria, making it exceptionally challenging to promote food safety culture in Nigeria. Although most of these informal food vendors provide cost-friendly alternative sources of food, the safety of foods sold in these places is substandard due to unhygienic ways of handling foods and a lack of sanitation, running water, refrigeration, and disinfection [17,30,34]. Sadly, the publics’ laissez-faire attitude to food safety measures, uncoordinated approach to food control, and poor enforcement of legislation and regulatory limits has exacerbated issues related to food safety practices and foodborne toxicants in Nigeria [73]. Furthermore, an inefficient food supply chain, poor traceability, and a lack of understanding of food safety and quality standards have contributed to these challenges [77]. The lack of data on the incidence of foodborne disease outbreaks in Nigeria and lack of awareness of the socioeconomic consequences of food safety issues have not helped either [38,60].

Other key obstacles to food safety in Nigeria include the lack of a positive attitude towards risk management and an unwillingness to learn from accidents, near misses, and safety performance indicators [78,79,80,81]. As reported in several studies, foods are often prepared in unsanitary conditions, regular washing of hands is somewhat rare, and foods are often exposed to flies and other insects [18,35,36,37,38,64,67,73,77,81]. Among certain ethnic groups, keeping and preparing foods in advance for consumption is a common practice, while in some communities, food poisoning is often associated with evil spirits and ancestral curses [82]. All of these fundamental challenges have crippled efforts to promote food safety practices among Nigerians [60], and the relaxed attitudes of the consuming public and the regulators have gained worrying traction. Efforts to overcome these challenges with an intensive public education campaign on food safety have also not yielded the expected results, because most food handlers and consumers preferred their cultural food safety practices passed on to them through previous generations. In addition, climate change presents an emerging threat to global food safety and security [83,84,85] and will also negatively impact consumers in Nigeria. Therefore, there is a great need to employ adaptive strategies, such as establishing a food safety management program in Nigeria which would expound on the need to detect food hazards and promote food safety culture. A starting point may be for the government to show more regulatory oversight. There is a consensus that regulatory oversight and better relationships with food producers could improve the current food safety deficiencies in Nigeria [86]. The government should constantly review food safety elements such as leadership, communication, risk perception, self-commitment, and management support.

## 7. Conclusions

The existing literature on food safety culture in Nigeria paints an incomplete picture of a problem that needs to be addressed. Most research has focused on the knowledge, attitude, and practices of food safety among food businesses (both informal and formal) in Nigeria. More research is needed to answer specific questions such as: What is the consumer demand for food safety in Nigeria? Does the demand for food safety in Nigeria differ between rural and urban consumers? Can consumers correctly discover food safety risks? What type of quality/safety certifications or food safety testing in Nigeria are considered more reliable by consumers? How do demands for food safety change throughout economic development and underdevelopment? How and to what extent does consumer demand get transmitted to producers, and who are the key players that influence this transmission? Are farmers and food businesses willing to change and improve their practices in response to consumer demand? Has the National Agency for Food and Drug Administration and Control (NAFDAC) been living up to the aims and objectives of its establishment? While these questions may seem complex, they are not insurmountable. Yet, adequately conveying food safety information to consumers and producers creates demands that not all consumers are willing to meet or that producers are willing to accept. Therefore, creating or improving the existing culture of food safety in Nigeria requires applying the best management and communication approaches in different regions and understanding local food safety practices. Without a doubt, a strong commitment by consumers, stakeholders, food businesses, and food handlers is needed to achieve this goal.

## Data Availability

Not applicable.

## References

[1] De Boeck E., Jacxsens L., Vanoverberghe P., Vlerick P. (2019). Method triangulation to assess different aspects of food safety culture in food service operations. Food Res. Int..

[2] Fontannaz-Aujoulat F., Frost M., Schlundt J. (2019). WHO Five Keys to Safer Food communication campaign-Evidence-based simple messages with a global impact. Food Control.

[3] Griffith C.J., Livesey K.M., Clayton D.A. (2010). Food safety culture: The evolution of an emerging risk factor?. Br. Food J..

[4] Nwankwo E., Agbasiere D. (2021). Who is right? Examining the state of safety in selected medium-class hotels in Nigeria. J. Hosp..

[5] Sharman N., Wallace C.A., Jespersen L. (2020). Terminology and the understanding of culture, climate, and behavioural change–Impact of organisational and human factors on food safety management. Trends Food Sci. Technol..

[6] Jespersen L., Griffiths M., Wallace C.A. (2017). Comparative analysis of existing food safety culture evaluation systems. Food Control.

[7] de Andrade M.L., Stedefeldt E., Zanin L.M., da Cunha D.T. (2020). Food safety culture in food services with different degrees of risk for foodborne diseases in Brazil. Food Control.

[8] Zanin L.M., Stedefeldt E., da Silva S.M., da Cunha D.T., Luning P.A. (2021). Influence of educational actions on transitioning of food safety culture in a food service context: Part 2-Effectiveness of educational actions in a longitudinal study. Food Control.

[9] Jespersen L., Butts J., Holler G., Taylor J., Harlan D., Griffiths M., Wallace C.A. (2019). The impact of maturing food safety culture and a pathway to economic gain. Food Control.

[10] de Andrade M.L., Stedefeldt E., Zanin L.M., Zanetta L.D.A., da Cunha D.T. (2021). Unveiling the food safety climate’s paths to adequate food handling in the hospitality industry in Brazil. Int. J. Contemp. Hosp. Manag..

[11] European Food Safety Authority and European Centre for Disease Prevention and Control (EFSA and ECDC) (2018). The European Union summary report on trends and sources of zoonoses, zoonotic agents and food-borne outbreaks in 2017. EFSa J..

[12] Griffith C.J. (2006). Food safety: Where from and where to?. Br. Food J..

[13] Nayak R., Waterson P. (2017). The Assessment of Food Safety Culture: An investigation of current challenges, barriers and future opportunities within the food industry. Food Control.

[14] Griffith C.J., Livesey K.M., Clayton D. (2010). The assessment of food safety culture. Br. Food J..

[15] Powell D.A., Jacob C.J., Chapman B.J. (2011). Enhancing food safety culture to reduce rates of foodborne illness. Food Control.

[16] Yiannas F. (2008). Food Safety Culture: Creating a Behavior-Based Food Safety Management System.

[17] Ezirigwe J. (2018). Much ado about food safety regulation in Nigeria. J. Sustain. Dev. Law Policy.

[18] Fasoro A., Faeji C., Oni O., Oluwadare T. (2016). Assessment of food safety practices in a rural community in Southwest Nigeria. Food Public Health.

[19] Ortega D.L., Tschirley D.L. (2017). Demand for food safety in emerging and developing countries: A research agenda for Asia and Sub-Saharan Africa. J. Agribus. Dev. Emerg. Econ..

[20] Amolegbe K.B., Upton J., Bageant E., Blom S. (2021). Food price volatility and household food security: Evidence from Nigeria. Food Policy.

[21] Aworh O.C. (2020). Food safety issues in fresh produce supply chain with particular reference to sub-Saharan Africa. Food Control.

[22] Oranusi S., Onyike E., Galadima M., Umoh V. (2004). Hazard Analyses Critical Control Points of foods Prepared By Families in Zaria Nigeria. Niger. J. Microbiol..

[23] Egbule O.S., Iweriebor B.C., Odum E.I. (2021). Beta-Lactamase-Producing Escherichia coli Isolates Recovered from Pig Handlers in Retail Shops and Abattoirs in Selected Localities in Southern Nigeria: Implications for Public Health. Antibiotics.

[24] Ifiora G.C., Chukwunwejim C.R., Ejikeugwu C.P., Egbuna R.N., Ifiora F.C., Abonyi I.C., Eze P.M., Arzai A.H., Mukhtar M.D. (2020). Microbiological safety assessment of food handlers in Wudil Local Government Area of Kano State, Nigeria. MicroMedicine.

[25] Anyogu A., Olukorede A., Anumudu C., Onyeaka H., Areo E., Adewale O., Odimba J.N., Nwaiwu O. (2021). Microorganisms and food safety risks associated with indigenous fermented foods from Africa. Food Control.

[26] Daniels N.A., Mackinnon L., Rowe S.M., Bean N.H., Griffin P.M., Mead P.S. (2002). Foodborne disease outbreaks in United States schools. Pediatr. Infect. Dis. J..

[27] Hedberg C.W., Smith S.J., Kirkland E., Radke V., Jones T.F., Selman C.A., Group E.-N.W. (2006). Systematic environmental evaluations to identify food safety differences between outbreak and nonoutbreak restaurants. J. Food Prot..

[28] Onyeaka H., Agbugba I., Ekwebelem O., Anumudu C., Anyogu A., Odeyemi O., Agbagwa S. (2021). Strategies to Mitigate the Impact of COVID-19 on Food Security and Malnutrition in Nigeria. Eur. J. Nutr. Food Saf..

[29] Ekwebelem O.C., Ofielu E.S., Nnorom-Dike O.V., Iweha C., Ekwebelem N.C., Obi B.C., Ugbede-Ojo S.E. (2020). Threats of COVID-19 to Achieving United Nations Sustainable Development Goals in Africa. Am. J. Trop. Med. Hyg..

[30] Iyadi R.C. (2015). Consumers’ Perception of Safety of Food In South–South And South–East of Nigeria. Ph.D. Thesis.

[31] Ogundugbe S. (2011). Ensuring Food Safety and Environmental Hygiene. J. Food Saf. Hyg..

[32] Isaac Adewole Food Safety as an Important Global Public Health and Economic Concern. http://healthnewsng.com/food-safety-important-global-public-health-economic-concern/.

[33] Kundu S., Banna M.H.A., Sayeed A., Akter S., Aktar A., Islam M.A., Proshad R., Khan M.S.I. (2021). Effect of vendors’ socio-demography and other factors on hygienic practices of street food shops. J. Foodserv. Bus. Res..

[34] Okojie P.W., Isah E.C. (2019). Food hygiene knowledge and practices of street food vendors in Benin City, Nigeria. Int. J. Consum. Stud..

[35] Gali A., Umaru G., Adamu S., Hamza I., Jibrin M. (2020). Assessment of operational facilities and sanitary practices in Zangon Shanu abattoir, Sabon Gari Local Government Area, Kaduna State, Nigeria. J. Vet. Med. Anim. Health.

[36] Abejegah C., Abah S., Awunor N., Duru C., Eluromma E., Aigbiremolen A., Okoh E. (2013). Market sanitation: A case study of Oregbeni market Benin-city Edo State, Nigeria. Int. J. Basic Appl. Innov. Res..

[37] Adeolu A., Opasola A., Salami O., Iyanda A., Omenta R. (2019). Sanitary Status and Compliance with the Standard Slaughter Practices in Karu Abattoir Abuja Municipal Area Council of the FCT, Nigeria. Int. J. Curr. Innov. Adv. Res..

[38] Onyeneho S.N., Hedberg C.W. (2013). An assessment of food safety needs of restaurants in Owerri, Imo State, Nigeria. Int. J. Environ. Res. Public Health.

[39] Ojo A.B. (2011). Consumers Attitude Towards Made in Nigeria and Foreign Made Foods. J. Food Sci. Technol..

[40] Ogunniyi A.I., Omotoso S.O., Salman K.K., Omotayo A.O., Olagunju K.O., Aremu A.O. (2021). Socio-economic Drivers of Food Security among Rural Households in Nigeria: Evidence from Smallholder Maize Farmers. Soc. Indic. Res..

[41] Ayeni K.I., Atanda O.O., Krska R., Ezekiel C.N. (2021). Present status and future perspectives of grain drying and storage practices as a means to reduce mycotoxin exposure in Nigeria. Food Control.

[42] Oko A., Ugwu S. (2011). The proximate and mineral compositions of five major rice varieties in Abakaliki, South-Eastern Nigeria. Int. J. Plant Physiol. Biochem..

[43] Nwali A.C., Maureen A. (2019). Marketing Analysis of Locally Produced Rice in Abakaliki Local Government Area of Ebonyi State Nigeria. Mediterr. J. Soc. Sci..

[44] David A., Adepoju P. The Dangerous Meteoric Rise of Artificial Fruit Ripening in Nigeria. http://www.healthnews.ng/the-dangerous-meteoric-rice-of-artificial-fruit-ripening-in-nigeria/.

[45] Matemilola S. (2017). The challenges of food security in Nigeria. Open Access Lib. J..

[46] Okoh N. (2011). Food Qualities and Supply Management. J. Food Sci. Technol..

[47] Udoh E., Udoh E. (2020). Food Safety Practices of Household Food Preparers in Akwa Ibom State, Nigeria. Adv. Soc. Sci. Res..

[48] Okpiaifo G., Durand-Morat A., West G.H., Nalley L.L., Nayga R.M., Wailes E.J. (2020). Consumers’ preferences for sustainable rice practices in Nigeria. Glob. Food Secur..

[49] Ezekiel C.N., Oyedele O.A., Kraak B., Ayeni K.I., Sulyok M., Houbraken J., Krska R. (2020). Fungal diversity and mycotoxins in low moisture content ready-to-eat foods in Nigeria. Front. Microbiol..

[50] Saharareporters 10 Dead, 400 Hospitalised after Drinking Juice in Kano. http://saharareporters.com/2021/04/15/10-dead-400-hospitalised-after-drinking-juice-kano.

[51] Saharareporters over 20 Dead after Eating ‘Fried Meat’ Served by Yahoo Boy at a Bar in Ogun. http://saharareporters.com/2021/03/29/over-20-dead-after-eating-%E2%80%98fried-meat%E2%80%99-served-yahoo-boy-bar-ogun.

[52] Olufemi A. Plateau Community Head, Others Suffer Food Poisoning Allegedly Caused by Looted Palliatives. https://www.premiumtimesng.com/regional/north-central/427604-plateau-community-head-others-suffer-food-poisoning-allegedly-caused-by-looted-palliatives.html.

[53] Nation T. Dangerous Meat, Toxic Waters 3: Nigerians Die from ‘Food Poisoning’. https://thenationonlineng.net/dangerous-meat-toxic-waters-3-nigerians-die-food-poisoning/.

[54] Africanews 13 Die of Food Poisoning in Nigeria. https://dailytrust.com/update-13-people-die-of-food-poisoning-in-abuja.

[55] Mehdizadeh Gohari I., Navarro M.A., Li J., Shrestha A., Uzal F., McClane B.A. (2021). Pathogenicity and virulence of Clostridium perfringens. Virulence.

[56] Times P. 26 Persons Survive Food Poisoning in Katsina. https://www.premiumtimesng.com/news/5436-26_persons_survive_food_poisoning_in_katsina.html.

[57] Adegbola J., Bamishaiye E., Olayemi F. (2011). Merchants’ attitude towards the use, and ban of the pesticide Gammalin in Dawanau International grain market, Kano, Nigeria. Adv. Bio Res..

[58] Hopkins M. Nigeria Bans 30 Pesticides after Deaths. AgriBusiness Global. https://www.agribusinessglobal.com/markets/africa-middle-east/nigeria-bans-30-pesticides-after-deaths/.

[59] Adeyemi B.A., Adeyemi B.B., Omiyefa O.M., Oyekan O.A. (2019). Diversity in religion and culture in nigeria: A blessing or a curse?. J. Pedagog. Thought.

[60] Kilders V., Caputo V., Liverpool-Tasie L.S.O. (2021). Consumer ethnocentric behavior and food choices in developing countries: The case of Nigeria. Food Policy.

[61] Idiaye C.O., Ogidan O.A., Oluwatayo I.B. (2020). Perception, risk attitude and willingness to pay for safety and innovative attributes of processed chicken meat in Oyo State, Nigeria. Ital. J. Food Saf..

[62] Akinbode S., Dipeolu A., Okuneye P. (2011). Willingness to pay for street food safety in Ogun State, Nigeria. J. Agric. Food Inf..

[63] Alimi B., Oyeyinka A., Olohungbebe L. (2016). Socio-economic characteristics and willingness of consumers to pay for the safety of fura de nunu in Ilorin, Nigeria. Qual. Assur. Saf. Crops Foods.

[64] Iwu A.C., Uwakwe K.A., Duru C.B., Diwe K.C., Chineke H.N., Merenu I.A., Oluoha U.R., Madubueze U.C., Ndukwu E., Ohale I. (2017). Knowledge, attitude and practices of food hygiene among food vendors in Owerri, Imo State, Nigeria. Int. J. Occup. Med. Environ. Health.

[65] Pepple N. (2017). Environment and food poisoning: Food safety knowledge and practice among food vendors in Garki, Abuja–Nigeria. J. Health Educ. Res. Dev..

[66] Chukwuocha U., Dozie I., Amadi A., Nwankwo B., Ukaga C., Aguwa O., Abanobi O., Nwoke E. (2009). The knowledge, attitude and practices of food handlers in food sanitation in a metropolis in south eastern Nigeria. East Afr. J. Public Health.

[67] Ehiri J.E., Azubuike M.C., Ubbaonu C.N., Anyanwu E.C., Ibe K.M., Ogbonna M.O. (2001). Critical control points of complementary food preparation and handling in eastern Nigeria. Bull. World Health Organ..

[68] Ifeadike C., Ironkwe O., Adogu P., Nnebue C., Emelumadu O., Nwabueze S., Ubajaka C. (2012). Prevalence and pattern of bacteria and intestinal parasites among food handlers in the Federal Capital Territory of Nigeria. J. Niger. Med. Assoc..

[69] da Cunha D.T. (2021). Improving food safety practices in the foodservice industry. Curr. Opin. Food Sci..

[70] Varrella S. Literacy Rate in Nigeria 2018, by Zone and Gender. https://www.statista.com/statistics/1124745/literacy-rate-in-nigeria-by-zone-and-gender/.

[71] Elom M.O., Eze U.A., Nworie A., Akpotomi I.O. (2012). Prevalence of geohelminths on edible fruits and vegetables cultivated in. Am. J. Food. Nutr..

[72] Ehuwa O., Jaiswal A.K., Jaiswal S. (2021). Salmonella, Food Safety and Food Handling Practices. Foods.

[73] Omojokun J. (2013). Regulation and enforcement of legislation on food safety in Nigeria. Mycotoxin Food Saf. Dev. Ctries..

[74] Federal of Ministry of Health (2014). National Policy on Food Safety and Its Implementation Strategy. http://extwprlegs1.fao.org/docs/pdf/nig151436.pdf.

[75] Nyor J. (2014). The role of regulatory agencies in food quality control in Nigeria. SCSR J. Agribus..

[76] NAFDAC (2019). Food Hygiene Regulations. https://www.nafdac.gov.ng/wp-content/uploads/Files/Resources/Guidelines/FOOD_GUIDELINES/Food-Hygiene-Guidelines.pdf.

[77] Global Alliance for Improved Nutrition (2020). Nigeria Policy, Monitoring Systems Analysis and Stakeholder Mapping Report. A USAID EatSafe Project Report. https://agrilinks.org/sites/default/files/media/file/Report%20on%20Policy%20and%20Monitoring%20Systems%20Analysis%20and%20Stakeholder%20Mapping-FINAL.pdf.

[78] Chikaire U., Atoma C., Oyem A., Akeni T. (2020). Displaced Farmers Perception of Resource-Use Conflicts as an Obstacle to Household Food Security and Food Safety in Abia State, Nigeria. J. Commun. Commun. Res..

[79] Ajayi O.A., Salaudeen T. (2014). Consumer food safety awareness and knowledge in Nigeria. Int. J. Food Saf..

[80] Odipe O.E., Raimi M., Deinkuro N.S., Funmilayo A.A., innocent Edewor O.-P., Lateefat H.M., Fadeyibi M. (2019). Assessment of Environmental Sanitation, Food Safety Knowledge, Handling Practice among Food Handlers of Bukateria Complexes in Iju Town, Akure North of Ondo-State, Nigeria. Act. Sci. Nutr. Health.

[81] Okpala C.O.R., Nwobi O.C., Korzeniowska M. (2021). Assessing Nigerian Butchers’ Knowledge and Perception of Good Hygiene and Storage Practices: A Cattle Slaughterhouse Case Analysis. Foods.

[82] Oyemade A., Omokhodion F.O., Olawuyi J.F., Sridhar M.K., Olaseha I.O. (1998). Environmental and personal hygiene practices: Risk factors for diarrhoea among children of Nigerian market women. J. Diarrhoeal Dis. Res..

[83] Oyedele O.A., Akinyemi M.O., Kovač T., Eze U.A., Ezekiel C.N. (2020). Food safety in the face of climate change: Consequences for consumers. Croat. J. Food Sci. Technol..

[84] Yakubu S.M., Musa M.W., Bamidele T.E., Ali M.B., Bappah M.T., Munir R.T., Manuwa A. (2020). Effects of Farmer-Herder Conflicts on Rural Households Food Security in Gombe State, Nigeria. J. Agric. Ext. Rural. Dev..

[85] Aluko O.I. (2020). Agricultural policy and food security in Nigeria: A rational choice analysis. The Palgrave Handbook of Agricultural and Rural Development in Africa.

[86] Nwaiwu O., Itumoh M. (2017). Chemical contaminants associated with palm wine from Nigeria are potential food safety hazards. Beverages.

